# Algoritmos Artificiais Superam os Modelos Tradicionais na Predição de Doença Arterial Coronariana

**DOI:** 10.36660/abc.20210823

**Published:** 2021-11-22

**Affiliations:** 1 Adiyaman Universitesi Egitim ve Arastirma Hastanesi Cardiology Adıyaman Turquia Adiyaman Universitesi Egitim ve Arastirma Hastanesi – Cardiology, Adıyaman - Turquia; 2 Sanko University – Cardiology Cardiology Gaziantep Turquia Sanko University – Cardiology, Gaziantep – Turquia

**Keywords:** Doença da Artéria Coronariana, Inteligência Artificial, Algorítmos, Angiografia da Coronária Computadorizada/métodos, Aprendizado de Máquina, Aprendizado Profundo, Máquina de Vetores de Suporte

Recomendações clínicas recentes indicam que testes adicionais para avaliar aspectos anatômicos (extensão, gravidade, morfologia) ou funcionais (função ventricular, presença/extensão da isquemia) da doença arterial coronariana (DAC) crônica e sintomática podem ser úteis em certos casos.^1^ Médicos do setor de emergência devem determinar se devem liberar o paciente, fazer mais testes não invasivos ou realizar angiografia invasiva em pacientes com desconforto torácico agudo. Aceitar qualquer pessoa com dor torácica pode ter efeitos indesejados em caso de alta com doença coronariana instável.^2^ A probabilidade de DAC obstrutiva deve orientar as decisões médicas.^3^ Algoritmos de aprendizado de máquina (*machine learning* — ML) podem complementar as capacidades diagnósticas e prognósticas dos métodos de regressão convencionais. A disparidade entre a aplicabilidade de tais métodos e os resultados alcançados com eles se deve às plataformas de software de análise de dados utilizadas.^4^

O ML pode usar recursos de sinal de fase torácica para construir modelos matemáticos finais que avaliem a existência de DAC importante. A análise do espaço da fase cardíaca parece semelhante aos testes de estresse funcional mais amplamente utilizados e requer pouco tempo do paciente.^5^ Os resultados de 2 anos mostraram que a reserva de fluxo fracionada derivada da TC por *deep learning* (DL-FFRCT) pode ser usada para guiar a revascularização, com alta taxa de cancelamento e baixa taxa de eventos. Uma DL-FFRCT positiva para lesões em tandem mostrou-se associada à redução de eventos cardiovasculares adversos maiores (ECAM) após 2 anos.^6^ O escore de risco isquêmico por ML (ML-IRS) obtido pela angiotomografia coronariana quantitativa reforçou a predição de revascularização futura e pode ser usado para identificar indivíduos com probabilidade de precisar de revascularização se encaminhados para cateterismo cardíaco. Esse escore de aprendizado de máquina está vinculado a medidas invasivas de reserva de fluxo fracionada (FFR), fornecendo validação externa entre dois centros e aumentando os modelos de predição de risco clínico.^7^

Mesmo com aparelhos de tomografia computadorizada (TC) mais antigos, a nova versão de reserva de fluxo fracionada derivada da TC (FFRCT) demonstrou excelente desempenho diagnóstico para lesões coronárias obstrutivas limitantes de fluxo, com uma redução substancial nos casos de falsos positivos, podendo reduzir o número de pacientes encaminhados para mais testes. O significado clínico desses resultados deve ser confirmado por pesquisas que avaliem os resultados clínicos. Esse programa utiliza tecnologias avançadas de aprendizado de máquina para melhorar a acessibilidade, a velocidade e para economizar custos.^8^

Al’Aref et al.,^9^ verificaram que a inteligência artificial (IA) alterou elementos fundamentais da existência humana. O ML, um tipo de IA em que os computadores aprendem o conhecimento de forma autônoma, identificando padrões a partir de enormes conjuntos de dados, é amplamente utilizado na medicina, principalmente em doenças cardiovasculares. Apresenta-se Uma breve introdução aos métodos de ML para a construção de modelos baseados em dados inferenciais e preditivos. Em particular, eles enfatizam técnicas de imagem não invasivas, como o escore de cálcio na artéria coronária e angiotomografia coronariana (ATC). No final do estudo, eles discutem as limitações atuais dos algoritmos de ML na área de doenças cardiovasculares.

Devido à sua capacidade de auxiliar na tomada de decisões e melhorar o desempenho diagnóstico e prognóstico, a IA está promovendo uma grande mudança de paradigma em diversas especialidades médicas, principalmente na Cardiologia. Apresenta-se aqui uma visão geral não sistemática dos principais artigos publicados sobre IA na área de Cardiologia, com foco em suas principais aplicações, efeitos e dificuldades.^10^ Apesar de apresentar melhores desfechos, a reserva de fluxo fracionada (FFR) permanece subutilizada na prática diária. Roguin et al.,^11^ pretendiam verificar se um programa de FFR baseado em angiografia por IA automatizada (AutocathFFR) pode ajudar os cardiologistas intervencionistas na tomada de decisões. O AutocathFFR foi utilizado para tirar fotos angiográficas de pacientes submetidos a medições de FFR com fio de pressão. O ponto de corte FFR 0,8 foi computado como sensibilidade e especificidade. A identificação automática da lesão funcionou em todas as lesões com FFR 0,8 ou inferior. Um FFR medido por fio de pressão >0,8 foi previsto com nível de precisão de 90% e uma área sob a curva de 0,91 pelo AutocathFFR. O AutocathFFR é uma tecnologia promissora que pode ajudar as pessoas com doença arterial coronariana a tomar melhores decisões e escolher melhores opções de tratamento.

A IA tem crescido constantemente devido aos avanços tecnológicos. Para melhorar a qualidade da coleta e reconstrução de imagens integrando as informações obtidas das imagens para construir modelos de predição poderosos, muitos algoritmos de IA têm sido usados para a DAC. Na angiotomografia, a IA pode ajudar em muitos aspectos de análise de placa, incluindo o grau de estenose e o formato da placa. Um número crescente de dados vincula algumas placas, denominadas placas de alto risco ou suscetíveis, a eventos cardiovasculares, independentemente da estenose. O radiologista deve compreender e se envolver ativamente no desenvolvimento e implementação da IA. Discutimos os méritos, limites, novas aplicações e possíveis avanços do uso de IA para caracterizar placas usando TC nesta revisão de literatura.^12^
