# Supplementary material for: Algoritmos Artificiais Superam os Modelos Tradicionais na Predição de Doença Arterial Coronariana
Source: Arq Bras Cardiol. 2021 Nov 22;117(6):1071–2. [Article in Portuguese] doi: 10.36660/abc.20210823 (PMC8757158; doi:10.36660/abc.20210823)
Supplement: Translation

# Translation: Artificial Algorithms Outperform Traditional Models in Predicting Coronary Artery Disease

**Affiliations:** 1 Adıyaman Turkey Adiyaman Universitesi Egitim ve Arastirma Hastanesi – Cardiology, Adıyaman - Turkey; 2 Gaziantep Turkey Sanko University – Cardiology, Gaziantep – Turkey

**Keywords:** Coronary Artery Disease, Artificial Intelligence, Algorithms, Angiography Coronary CT/methods, Machine Learning, Deep Learning, Support Vector Machine

Recent clinical recommendations indicate that additional tests for assessing anatomical (extent, severity, morphology) or functional (ventricular function, presence/extent of ischemia) aspects of chronic and symptomatic coronary artery disease (CAD) may be helpful in certain cases.^1^ Emergency physicians must determine whether to release the patient, do further non-invasive testing, or perform invasive angiography on patients with acute chest discomfort. Accepting anybody with chest pain may have unintended effects if discharged with unstable coronary disease.^2^ The likelihood of obstructive CAD should guide medical decisions.^3^ Machine learning (ML) algorithms can supplement the diagnostic and prognostic capabilities of conventional regression methods. The disparity between the applicability of such methods and the outcomes achieved with them was due to the data analysis software platforms used.^4^

ML may use thoracic phase signal features to build final mathematical models that evaluate the existence of severe CAD. Cardiac phase space analysis seems similar to the most widely used functional stress tests and needs little patient time.^5^ The 2-year results showed that deep learning fractional flow reserve derived from CT (DL-FFRCT) may be used to guide revascularization, with high cancellation rate and low event rate. A positive DL-FFRCT for tandem lesions was linked with reduced major adverse cardiac events (MACEs) after 2 years.^6^ The ML-ischemia risk score (ML-IRS) obtained from quantitative coronary CT angiography enhanced the prediction of future revascularization and may be used to identify individuals who are likely to need revascularization if referred for cardiac catheterization. This machine learning score is linked with invasive fractional flow reserve (FFR) measures, providing external validation across two centers and augmenting clinical risk prediction models.^7^

Even with older computed tomography (CT) scanners, the new version of fractional flow reserve derived from CT (FFRCT) demonstrated excellent diagnostic performance for flow-limiting obstructive coronary lesions, with a substantial reduction in false-positive instances, which may reduce the number of patients referred for further testing. The clinical significance of these results must be confirmed by research evaluating clinical outcomes. This program uses cutting-edge machine learning technologies to improve accessibility, speed, and save costs.^8^

Al’Aref et al.^9^ found out that artificial intelligence (AI) has changed fundamental elements of human existence. ML, a type of AI in which computers autonomously learn knowledge by identifying patterns from huge datasets, is widely used in medicine, particularly in cardiovascular disease. A short introduction of ML methods for building inferential and predictive data-driven models is presented. In particular, they emphasize non-invasive imaging techniques such as coronary artery calcium scoring and coronary computed tomography angiography (CTA). In the end of their study, they discuss the current limitations of ML algorithms in the field of cardiovascular illness.

Because of its capacity to assist decision-making and improve diagnostic and prognostic performance, AI is promoting a major paradigm change in a wide range of medical fields, especially in Cardiology. A non-systematic overview of the main articles published on AI in Cardiology is presented here, focusing on its primary applications, effects, and difficulties.^10^ Despite better patient outcomes, fractional flow reserve (FFR) remains underused in daily practice. Roguin et al.^11^ wanted to see whether an automated AI angiography-based FFR program (AutocathFFR) may help interventional cardiologists make decisions. AutocathFFR was used to take angiographic pictures of patients who had pressure wire FFR measurements. The FFR cut-off 0.8 was computed sensitivity and specificity. Automatic lesion identification worked on all lesions with FFR 0.8 or below. A wire-based FFR >0.8 was predicted with accuracy level of 90% and an area under the curve of 0.91 by AutocathFFR. AutocathFFR is a promising technology that may help people with coronary artery disease make better decisions and choose better treatment options.

AI has grown steadily owing to technological advancements. To enhance the quality of picture collection and reconstruction while integrating information obtained from the images to build powerful prediction models, many AI algorithms have been used for CAD. In CTA, AI can help with many aspects of plaque analysis, including stenosis degree and plaque shape. An increasing body of data links some plaques, termed high-risk or susceptible plaques, to cardiovascular events, regardless of stenosis. The radiologist must understand and actively engage in the development and implementation of AI. We discuss the merits, limits, new applications, and potential advancements of using AI to characterize plaques using CT in this current literature review.^12^
